# Detecting agitation and aggression in persons living with dementia: a systematic review of diagnostic accuracy

**DOI:** 10.1186/s12877-024-05143-6

**Published:** 2024-06-26

**Authors:** Britney Wong, Pauline Wu, Zahinoor Ismail, Jennifer Watt, Zahra Goodarzi

**Affiliations:** 1 Department of Community Health Sciences, University of Calgary, Calgary, Canada; 2 Department of Medicine, University of Calgary and Alberta Health Services, Calgary, Canada; 3 Hotchkiss Brain Institute, University of Calgary, Calgary, AB Canada; 4 Department of Clinical Neurosciences, University of Calgary, Calgary, AB Canada; 5 Department of Psychiatry, University of Calgary, Calgary, AB Canada; 6 Cumming School of Medicine, University of Calgary, Calgary, AB Canada; 7 O’Brien Institute of Public Health, University of Calgary, Calgary, AB Canada; 8 Division of Geriatric Medicine, University of Toronto, Toronto, ON Canada; 9 Department of Medicine, University of Toronto, Toronto, ON Canada

**Keywords:** Agitation, Aggression, Dementia, Diagnostic accuracy

## Abstract

**Objective:**

40–60% of persons living with dementia (PLWD) experience agitation and/or aggression symptoms. There is a need to understand the best method to detect agitation and/or aggression in PLWD. We aimed to identify agitation and/or aggression tools that are validated against a reference standard within the context of PLWD.

**Methods:**

Our study was registered on PROSPERO (CRD42020156708). We searched MEDLINE, Embase, and PsycINFO up to April 22, 2024. There were no language or date restrictions. Studies were included if they used any tools or questionnaires for detecting either agitation or aggression compared to a reference standard among PLWD, or any studies that compared two or more agitation and/or aggression tools in the population. All screening and data extraction were done in duplicates. Study quality was assessed using the Quality Assessment of Diagnostic Accuracy Studies 2 (QUADAS-2) tool. Data extraction was completed in duplicates by two independent authors. We extracted demographic information, prevalence of agitation and/or aggression, and diagnostic accuracy measures. We also reported studies comparing the correlation between two or more agitation and/or aggression tools.

**Results:**

6961 articles were screened across databases. Six articles reporting diagnostic accuracy measures compared to a reference standard and 30 articles reporting correlation measurements between tools were included. The agitation domain of the Spanish NPI demonstrated the highest sensitivity (100%) against the agitation subsection of the Spanish CAMDEX. Single-study evidence was found for the diagnostic accuracy of commonly used agitation scales (BEHAVE-AD, NPI and CMAI).

**Conclusions:**

The agitation domain of the Spanish NPI, the NBRS, and the PAS demonstrated high sensitivities, and may be reasonable for clinical implementation. However, a limitation to this finding is that despite an extensive search, few studies with diagnostic accuracy measurements were identified. Ultimately, more research is needed to understand the diagnostic accuracy of agitation and/or aggression detection tools among PLWD.

**Supplementary Information:**

The online version contains supplementary material available at 10.1186/s12877-024-05143-6.

## Key points

• The agitation domain of the Spanish Neuropsychiatric Inventory (NPI), Neurobehavioural Rating Scale (NBRS), and the Pittsburgh Agitation Scale (PAS) demonstrated high sensitivities for agitation and may be reasonable for clinical implementation. However, many commonly used agitation tools have yet to be assessed for their diagnostic accuracy.

• Only one study described diagnostic accuracy measures for only aggression, with a moderate sensitivity reported.

• More rigorous studies are needed to understand the diagnostic accuracy of common agitation and/or aggression tools within the context of dementia.

**Supplementary Information:**

The online version contains supplementary material available at 10.1186/s12877-024-05143-6.

## Introduction

Dementia is a progressive neurodegenerative disorder characterized by cognitive and functional impairment [1]. Persons living with dementia (PLWD) commonly experience burdensome neuropsychiatric symptoms, including depression, anxiety, apathy, agitation and aggression [2]. These comorbid symptoms often go under-recognized, indicate impending cognitive decline, and are elusive to treat [3]. Of these symptoms, agitation and aggression are particularly common and distressing symptoms among PLWD, with an overall prevalence of 30% and 50% within the dementia population, respectively [4, 5]. This prevalence varies by the underlying pathology and severity of dementia [6].

In 2015, the International Psychogeriatric Association formally published a definition for agitation, as a syndrome that includes any type of excessive motor activity, verbal aggression, or physical aggression causing distress [7]. Aggression refers to verbal and physical behaviour (e.g., hitting, throwing, etc.) with the potential to harm one’s self or others [8, 9]. Despite being separate constructs, they often are presented together among PLWD. Ultimately, PLWD who are experiencing either agitation or aggression have a poorer quality of life, difficulty accomplishing their daily activities, and are more likely to be admitted to long-term care facilities [1]. Likewise, caregivers of PLWD experiencing co-existing agitation or aggression face higher caregiver burden, a higher risk for injuries, and poorer quality of life [8, 10].

Early and accurate detection of agitation and aggression is beneficial to identify the antecedent contributors either intrinsic or extrinsic, enable early intervention and prevent harm [4, 11]. A systematic review of all interventions for symptoms of agitation and/or aggression in PLWD identified a lack of consistency in tools used to measure these symptoms, thus awareness of tool validity can also inform research in this area [12]. Moreover, these tools must be taken in the context of the PLWD and surrounding factors including antecedent events, severity, and personal attributes [12]. Although many tools have been created and examined, there is a lack of diagnostic accuracy information (e.g. sensitivity and specificity) for these tools. Diagnostic accuracy (e.g. sensitivity and specificity) is considered the ability of a tool or test to discriminate between the presence and absence of a condition (i.e. agitation and aggression) as compared to a reference standard [13].

Until 2015, there lacked a consensus-based definition of agitation, and consequently a reference standard diagnosis [14]. The lack of definition resulted in challenges in formally validating currently used agitation and/or aggression tools outside of expert opinion as the reference standard, resulting in a knowledge gap around the diagnostic accuracy of agitation and aggression tools [15]. Watt et al. (2019) identified the Behavioral Pathology in Alzheimer’s Disease Rating Scale (BEHAVE-AD), Neuropsychiatric Inventory (NPI), and Cohen-Mansfield Agitation Inventory (CMAI) as the most commonly used agitation and/or aggression detection tools among randomized controlled trials (RCTs) [12]. Although many of these tools have established content validity in the literature [16], the diagnostic accuracy is unclear. Therefore, the objective of this systematic review is to determine which tools are validated for detecting agitation and/or aggression among PLWD, in any setting.

## Methods

The study protocol was created a priori, follows the methods of the Cochrane collaboration, and is reported as per the Preferred Reporting Items for Systematic Reviews and Meta-Analyses (PRISMA) Diagnostic Test Accuracy (DTA) standards and guidelines. This was registered on PROSPERO (CRD42020156708) [17]. The PRISMA DTA checklist is also provided for this study (Supplemental Appendix 10).

### Selection criteria

The population included persons with any type or severity of dementia in any setting (i.e. clinic, nursing home, etc.). In the literature, the majority of studies refer to both agitation and aggression together. Therefore, we looked for studies that used any tools or questionnaires for detecting either agitation or aggression (i.e. Cohen-Mansfield Agitation Inventory, etc.), or both. However, we considered agitation and aggression as separate constructs. Given that the criteria for agitation and/or aggression is variable across settings and locations, we included any relevant reference standard, including any healthcare provider’s diagnosis of agitation and/or aggression using standard criteria (i.e. IPA criteria), or a diagnosis by a physician with expert training, such as psychiatrists and/or geriatricians [18]. The specific healthcare providers considered for the reference standard included geriatricians, general practitioners, or any other certified medical doctor (MD) working in geriatric care. As a secondary objective, we included articles that compared between two or more agitation and/or aggression tools, to understand how agitation and/or aggression tools correlated with one another.

### Search strategy

The search strategy was created and refined alongside an experienced librarian (HLR) and experienced clinician scientists (Z.G, Z.I, J.W). The databases MEDLINE, Embase, and PsycINFO were searched from inception until April 22, 2024 (Supplemental Appendix 1). The main search clusters were “dementia terms”, “agitation and/or aggression terms” and “diagnostic accuracy terms”, and each cluster was combined using the term “and” (Supplemental Appendix 8). Within each main cluster, keywords and database-specific words were searched, with each combined using the term “or” (Supplemental Appendix 8). All types of dementia were included in the search. There were no language, age of patient, or year of publication restrictions placed on articles. A grey literature search was conducted until September 4th, 2021 (Supplemental Appendix 2). Grey literature included all literature not formally published in an academic journal or book, to ensure our search was the most exhaustive [19].

### Screening and eligibility

The abstract screening was completed after a calibration (with B.W, P.W, Z.G, J.W), by B.W and P.W. independently and in duplicates. All articles that discussed a group or sub-group of persons living with dementia and an agitation and/or aggression tool were included at the abstract stage. If any disagreement arose between authors at the first stage it was included to full text.

**Fig. 1:**
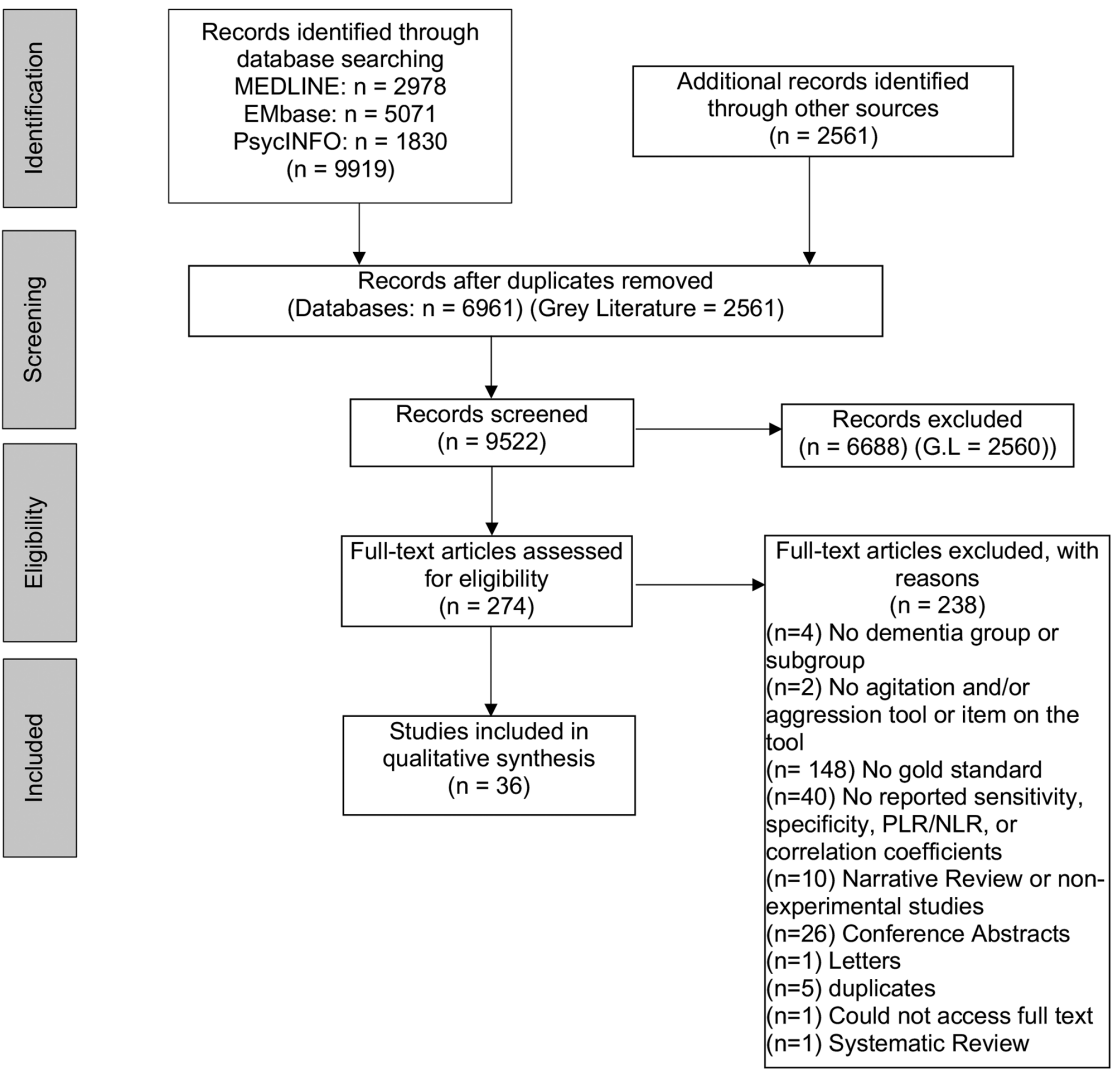
The PRISMA diagram [62] depicting the search and screening methodologies throughout the review

The full text screening process was calibrated between four authors (B.W, P.W, Z.G, J.W) and then screened in duplicates by the same independent authors (B.W, P.W). A list of exclusion criteria at the full text stage are reported in Fig. 1. All study designs except reviews, non-experimental studies, and letters were included. Two separate syntheses were conducted at the full text screening stage. Firstly, eligibility at the full-text stage required the use of a group or subgroup of persons living with any type of dementia, an agitation and/or aggression diagnostic tool, and a reference standard diagnosis of agitation and/or aggression. Studies were included for data extraction if they stated diagnostic accuracy measures of an agitation and/or aggression tool, against the reference standard. We defined diagnostic accuracy as the ability of the test to discriminate between agitation and/or aggression and lack thereof among PLWD [13]. We focused on measures of sensitivity, specificity, and positive and negative likelihood ratios as our outcomes of choice, given that we can best measure validity by comparing index tools against the reference standard diagnosis of agitation and/or aggression. We also considered positive and negative predictive values and the area under the ROC curve or minimum clinically important differences as additional diagnostic accuracy measures. Secondly, if a reference standard was not present, the article was searched for a comparison between two agitation and/or aggression tools to examine correlation coefficients as a secondary outcome and included in the final data extraction. This data was considered a measurement of construct validity, given that the tools we compared measured the same constructs of agitation and/or aggression. Included articles were verified between authors (B.W, P.W), with any discrepancies settled with an adjudicated third author (Z.G). As well, we screened the list of references for all included articles for any other potentially relevant articles. All non-English texts were translated with online translation software (Google Translate). Any French or Spanish articles were translated by a fluent speaker.

### Assessment of risk of bias

We assessed the quality of each included study with the Quality Assessment of Diagnostic Accuracy Studies 2 (QUADAS-2) tool by two independent authors (B.W, P.W) [20]. The completed Risk of Bias assessment was subsequently reviewed by an experienced clinician scientist (Z.G).

### Data extraction and synthesis of evidence

The data extraction form was developed by two authors (B.W, P.W) and verified by the experienced clinicians (Z.G, Z.I, J.W). Data extraction was conducted independently in duplicate (B.W, P.W). Demographic information and characterization of the type and severity of dementia were collected. The specific agitation and/or aggression tool and the reference standard were identified, along with respective agitation and/or aggression prevalence rates determined by either measure. Sensitivity and specificity values along with positive and negative likelihood ratios, and positive and negative predictive values were extracted. Finally, for studies focused on comparing two agitation and/or aggression tools, correlation coefficients were extracted as a secondary diagnostic accuracy measure along with the aforementioned demographic information.

## Results

### Database searches

The initial database searches yielded 9919 total results, and upon removal of duplicates, 6961 articles remained. The grey literature search found 2561 articles. There were 274 articles included for full-text screening (Fig. 1). After full-text screening, a total of 36 articles were included in the final data extraction stage. These articles are comprised of 6 articles reporting diagnostic accuracy measures compared to a reference standard, along with 30 articles reporting a comparison between tools. Given the low number of included articles reporting diagnostic accuracy measures, there was insufficient data for a meta-analysis.

### Summary of included studies comparing tools to a reference standard

Six studies were included that explored the diagnostic accuracy of agitation and/or aggression tools among PLWD compared to a reference standard [14, 15, 21–24]. One study reported diagnostic accuracy measures for only aggression [22], and five studies reported measures for only agitation [14, 15, 21, 23, 24]. They were published between 1999 and 2022, and conducted in Canada (*n* = 1), Spain (*n* = 1), France (*n* = 2), and the United States (*n* = 2) (Table 1) [14, 15, 21–24]. Sample sizes ranged from 30 to 19,424 participants [14, 15, 24]. The types of dementia included were Alzheimer’s Disease (*n* = 2), Vascular Dementia (*n* = 1), dementia with Lewy bodies (*n* = 2), mixed dementia (*n* = 1), probable Alzheimer’s disease (*n* = 1), frontotemporal dementia (*n* = 1), or unspecified dementia (*n* = 4) (Table 1) [14, 15, 21–24]. Dementia was diagnosed using the DSM [15, 21], DSM-IV-TR [22], the dementia diagnosis section of the CAMDEX [23] and DSM-III [24], with one study not reporting the method of diagnosis [14]. Dementia severity was assessed with the Mini Mental State Examination (MMSE) tool [14, 21, 22] and the dementia severity section of the CAMDEX [23]. Severity ranged from mild [14, 23] to severe [22], with three studies not reporting dementia severity [15, 24] (Table 1). The agitation and/or aggression tools used include the Empirical Behavioral Pathology in Alzheimer’s Disease Rating Scale (E-BEHAVE-AD) (*n* = 1), Neurobehavioural Rating Scale (NBRS) (*n* = 2), the agitation domain of the Neuropsychiatric Inventory (NPI, English and Spanish versions) (*n* = 3), the IPA definition of agitation constructed via items from the Neuropsychiatric Inventory Questionnaire (NPI-Q) (*n* = 1), French- Rating Scale for Aggressive Behaviour in the Elderly (F-RAGE) (*n* = 1), Pittsburgh Agitation Scale (PAS) (*n* = 1), Cohen Mansfield Agitation Inventory (CMAI) (*n* = 1), CMAI-IPA (*n* = 1) and NPI-C-IPA (*n* = 1) [14, 15, 21–24] (Supplemental Appendix 6). The reference standards were the Clinical Global Impression – Severity (CGI-S) scale (*n* = 1) [21], a subsection of the Spanish CAMDEX assessing agitation and/or aggression symptoms (*n* = 1) [23], the Alzheimer’s Disease Cooperative Study-Clinical Global Impression of Change (mADCS-CGIC) (*n* = 1) [15] or a psychiatrist’s or clinician’s diagnosis (*n* = 3) [14, 22, 24]. Vilalta-Franch et al.’s (1999) study was presented in Spanish, and was translated via Google Translate, whilst all other articles written in English [23]. The type and prevalence of agitation and/or aggression among studies comparing tools to a reference standard are reported in Table 2.

**Table 1:** Demographic information of included studies that compared agitation and/or aggression tools to a reference standard within a population of dementia

Author	Year	Agitation and/or Aggression Index Tool	Country	Setting	Mean Age of Participants	Total % Female	Total Sample Size	% Female in Dementia Subgroup	Dementia Subgroup size	Type of Dementia	Mild, Moderate or Severe?	Tools used for dementia diagnosis	Tools used for dementia severity	Score	Variance	Administrator of tool
Ismail et al.	2013	E-BEHAVE-AD, NBRS, NPI	Canada	Inpatient Units	81.2	58.6	87	58.6	87	AD, VD, dementia with Lewy bodies, mixed dementia, dementia not otherwise specified	Moderate to higher	DSM criteria	MMSE	9.7	62.41	Trained Research Assistants
Adama et al.	2013	F-RAGE	France	Hospital	83.3	63.29%	79	NR	36	NR	Severe	DSM-IV-TR	MMSE	17.9	50.41	Trained researcher
Vilalta-Franch et al.	1999	Spanish-NPI	Spain	Hospital	72.76	50.79%	63	NR	44	NR	Mild	CAMDEX	CAMDEX	NR	NR	Neurologist
Rosen et al.	1999	PAS, NBRS	USA	Clinics	> 60	NR	30	NR	30	NR	NR	DSM-III	NR	NR	NR	Clinician
Mauleon et al.	2020	CMAI, NPI C A + A, CMAI-IPA, NPI-C-IPA	France	Clinics and LTC facilities	82.4 +/- 7.2	58.40%	262	58.40%	262	Probable AD	NR	DSM criteria	MMSE	10	64	Rating clinicians
Sano et al.	2022	IPA Criteria (via NPI-Q items) ††	USA	Community	72.7	50.80%	19,424	NR	NR	AD, FTLD, LBD	NR	NR	MMSE	22.0	40.96	Rating clinicians

†**NR = Not Reported**

**††IPA criteria was constructed as an index tool using items from the Neuropsychiatric Inventory Questionnaire (NPI-Q)**

**Table 2:** Prevalence of agitation and/or aggression among PLWD in studies comparing tools to a reference standard*

Based on Index Tool						Based on Reference Standard						
Author	Year	Agitation and/or Aggression Index Tool	Type of Agitation		Cut-off for Agitation	Prevalence of agitation	Cut-off for Aggression	Type of Aggression	Prevalence of aggression	Gold Standard Tool/Method	Prevalence of agitation	Prevalence of aggression
Ismail et al.	2013	E-BEHAVE-AD	All		30%**	100%	30%	Physical	97.70%	CGI-S	NR†	
		NBRS	All		50%**	97.7%	50%	Physical	73.56%			
		NPI	All		50%**	95.4%	50%	Physical	NR†			
Adama et al.	2013	F-RAGE					≥ 8	Physical or Verbal	29%	Psychiatrist		31 patients (39.2%)
Vilalta-Franch et al.	1999	Spanish NPI	NR†		NR†	29%				CAMDEX	NR†	
Rosen et al.	1999	PAS	Mild agitation		A score of 4 on any item yields optimal SN, while 14–15 yields optimal SP	NR†				Psychiatrist based on IPA	NR†	
		NBRS	Mild agitation		60	NR†						
Mauleon et al.	2020	CMAI	All		NR†	23.7%				IPA definition of agitation	Excessive motor activity: 76.3%	Verbal: 76.3% Physical:44.1%
		NPI-C A + A	All		NR†	6.0%						
		CMAI-IPA	All		NR†	14.7%						
		NPI-C-IPA	All		NR†	5.8%						
Sano et al.	2022	IPA criteria (via NPI-Q items) ††	All		≥ 2 items fulfilled, or severity measure was ≥ 2 in one or more items	NR†				Clinician’s Diagnosis	15.7%	

†**NR = Not Reported**

††IPA criteria was constructed as an index tool using items from the Neuropsychiatric Inventory Questionnaire (NPI-Q)

*Grey squares indicates not applicable to the tool

**These cut-off values refer to the proportion of target symptoms resolved per patient at the identified sensitivity and specificity values (see Table 3**). The optimal cut-off points to maximize sensitivity and specificity are reported**

### Summary of tools

The NPI, NBRS and PAS are observational scales [21, 25]. The NPI is the main tool used for RCTs, with use reported among (*n* = 39) RCTs [12]. The NPI is a common informant-rated questionnaire used to assess neuropsychiatric symptoms in PLWD [26]. Within each of 12 domains, the informant is first asked a screening question for each neuropsychiatric symptom [27]. Should they initially indicate any problems in the agitation domain, the informant is then asked an additional 8 items in the agitation domain, with the frequency, severity, and distress of agitation calculated on Likert scales [28]. Only 1 domain is focused on agitation and/or aggression, and the overall tool is not focused solely on these symptoms.

Only one of 27 items on the NBRS focuses on assessing agitation [29]. Specifically, it assesses motor manifestations of overactivation [29]. Lastly, the PAS was developed to specifically examine agitation and/or aggression. It has 4 items assessing severity of agitation and/or aggression in four domains: aberrant vocalizations, motor agitation, aggressiveness, and resisting care [25]. The PAS is the only scale that solely analyzes agitation and/or aggression symptoms.

The BEHAVE-AD is a severity scale, used for dementia-related behavioural changes. It contains a global assessment of the overall magnitude of disturbance to the caregiver and patient due to the behavioural symptoms. The RAGE is an informant-rated scale that assesses verbal and physical aggression in institutionalized or hospitalized elderly patients.

### Outcomes of studies comparing tools with a reference standard (table 3)

**Table 3:** The sensitivity and specificity values of agitation and/or aggression diagnostic tools used within a dementia population among included studies that compared tools to a reference standard

Author	Year	Sensitivity of agitation tool	Sensitivity of aggression tool	Combined sensitivity of agitation and aggression tool	Specificity of agitation tool	Specificity of aggression tool	Combined specificity of agitation and aggression tool	PLR† of agitation tool	PLR of aggression tool	Combined PLR of agitation and aggression tool	NLR of agitation tool	NLR of aggression tool	Combined NLR of agitation and aggression tool	Minimal Clinically Important Difference (MCID) Scores
Ismail et al.	2013	E-BEHAVE-AD: 0.79		E-BEHAVE-AD: 0.79	E-BEHAVE-AD: 0.73		E-BEHAVE-AD: 0.73	E-BEHAVE-AD: 2.93		E-BEHAVE-AD: 2.93	E-BEHAVE-AD: 0.29		E-BEHAVE-AD: 0.288	N/A
		NBRS: 0.89		NBRS: 0.89	NBRS: 0.85		NBRS: 0.85	NBRS: 5.93		NBRS: 5.93	NBRS: 0.13		NBRS: 0.13	NR
		NPI: 0.86		NPI: 0.86	NPI: 0.76		NPI: 0.76	NPI: 3.58		NPI: 3.58	NPI: 0.18		NPI: 0.18	NR
Adama et al.	2013		F-RAGE: 0.74	0.74		0.98	0.98		37	37		0.27	0.27	NR
Vilalta-Franch et al.	1999	Spanish NPI: 1.00		1.00	0.98		0.98	44.84		44.84	0		0	NR
Rosen et al.	1999	PAS: 0.86 NBRS: 0.95		PAS: 0.86 NBRS: 0.95	PAS: 0.57 NBRS: 0.29		PAS: 0.57 NBRS: 0.29	NR		NR	NR		NR	NR
Mauleon et al.	2021	NR**††**	NR	NR	NR	NR	NR	NR	NR	NR	NR	NR	NR	One month: CMAI: -5 CMAI-IPA: -2 NPI-C: -3 NPI-C-IPA: -5 3 months: CMAI: -17 CMAI-IPA: -5 NPI-C: -3 NPI-C-IPA: -5
Sano et al.	2022	IPA criteria**: 0.79		IPA criteria: 0.79	0.69		0.69	2.55		2.55	0.30		0.30	N/A

*Grey squares indicates not applicable to the tool

**†PLR = positive likelihood ratio; NLR = negative likelihood ratio**

**††NR = Not Reported**

** The IPA criteria was constructed as an index tool using items from the Neuropsychiatric Inventory Questionnaire (NPI-Q)

Seven tools assessing agitation or aggression were identified that compared to a reference standard. Mauleon et al.’s (2021) study demonstrated the minimal clinically important difference (MCID) of the CMAI, agitation domain of the NPI-C, CMAI-IPA, and NPI-C-IPA [15]. The MCID, although not the same as sensitivity and specificity, represents an important construct. It identifies the minimal difference in score needed to show a beneficial change in symptoms as reported by a patient [30]. The MCID thus crucially identifies how useful a tool is for detecting clinically meaningful differences in agitation and/or aggression symptoms over time.

#### E-BEHAVE-AD

The E-BEHAVE-AD was evaluated for agitation detection by one study [21]. The sensitivity was 79.0% and specificity was 73.0%, compared to the CGI-S as the reference standard (Table 3). In the context of agitation, the CGI-S is an observer-rated instrument measuring the severity of agitation at one point in time, based on a clinician’s understanding of agitation in PLWD [21]. The positive likelihood ratio (PLR) and negative likelihood ratio (NLR) were 2.93 and 0.28, respectively.

#### NBRS

The NBRS was evaluated by two studies [21, 24] for agitation. Sensitivity ranged from 89.0 to 95.2%, whilst specificity ranged from 28.6 to 85.0% (Table 3). Ismail et al. (2013) used the CGI-S as the reference standard, while Rosen et al. (1999) used a psychiatrist’s diagnosis of agitation. Ismail et al., (2013) reported a PLR value of 5.93 and an NLR of 0.13 (Supplemental Appendix 9).

#### NPI

The agitation domain of the NPI was evaluated by a single study [21] for its ability to assess agitation. A sensitivity of 86.0% and specificity of 76.0% were obtained, compared to the CGI-S as a reference standard (Table 3). The PLR and NLR values reported were 3.58 and 0.18, respectively (Table 3). Mauleon et al., (2020) assessed the MCID of the agitation domain of the NPI-C, and the NPI-IPA against the mADCS-CGIC. They reported an MCID of -3 and − 5 for the NPI-C and NPI-IPA at one month, respectively [15]. These MCID scores mean that a clinically meaningful decline in agitation and/or aggression symptoms can be detected over a -3 and − 5 difference in scores when administered consecutively over 1 month, respectively. The MICD scores at 3 months were − 3 and − 5 for the NPI-C and NPI-C-IPA, respectively.

Sano et al. (2022) constructed the IPA definition of agitation using items 4 (agitation), 11 (motor disturbance) and 10 (irritability) of the Neuropsychiatric Inventory-Questionnaire (NPI-Q). They measured this construct against a clinician’s diagnosis of agitation as a reference standard. They reported a sensitivity of 79.0%, and a specificity of 69.0%. The PLR and NLR values were 2.55 and 0.30, respectively (Table 3).

#### Spanish NPI

The agitation domain of the Spanish NPI was used as a diagnostic tool for agitation, against the agitation subsection of the Spanish CAMDEX as a reference standard by one study [23]. A sensitivity of 100.0% and specificity of 97.8% were reported (Table 3). PLR and NLR values reported were 44.84 and 0.00, respectively (Supplemental Appendix 9).

#### PAS

The PAS was evaluated by one study to detect agitation, and was found to have a sensitivity of 85.7% and a specificity of 57.1%, when compared a psychiatrist’s diagnosis for agitation (Table 3) [24]. No PLR or NLR values were reported.

#### CMAI

The CMAI and CMAI-IPA were assessed in one study for their abilities to assess agitation, via MCID scores against the mADCS-CGIC [15]. They reported MCID scores of -5 and − 2 for the CMAI and CMAI-IPA at 1 month, respectively. These MCID scores mean that a clinically meaningful decline in agitation and/or aggression symptoms can be detected over a -5 and − 2 difference in scores when administered consecutively over 1 month, respectively. The MCID scores at 3 months were − 17 and − 5 for the CMAI and CMAI-IPA, respectively.

#### F-RAGE

The F-RAGE, was evaluated by a single study for physical and verbal aggression, demonstrated a sensitivity of 74.0%, and a specificity of 98.0% (Table 3) [22]. The reference standard was a psychiatrist’s diagnosis. The PLR was 37.00 and NLR was 0.26 (Supplemental Appendix 9).

### Summary of included studies comparing between tools (table 4)

**Table 4:** Demographic and validity measures of included studies that compared two or more agitation and/or aggression tools

Author	Year	Country	Setting		Mean Age	Total % Female	Total Sample Size	% Female Dementia Subgroup	Dementia Subgroup size	Type of Dementia	Mild, Moderate or Severe?	Tools used for dementia severity	Score	Variance	Tool Administrator	Tools being compared**	Correlation Coefficients	Agitation/ Aggression	Type of Coefficient
Whall et al.	2013	USA	Nursing homes		87.1	87.90%	107	87.90%	107	AD, VD	majority severe	MMSE	8.6	46.24	NR	CMAI and RAS-PABS	0.544	aggressive behaviour	Cohen’s Kappa
Choy et al.	2001	China	Clinic		80.48	63.92%	194	65.24%	164	AD, VD	NR	CMMSE	8.77	47.75	psychiatrist	C-BEHAVE-AD and CCMAI	0.76	aggressivity	Spearman’s
Cohen-Mansfield and et al.	2004	USA	Nursing homes		87	78.00%	175	78%	175	All	NR	MMSE	7	38.069	NR	CMAI and ABMI	PA: 0.411 OCA: 0.203	physical aggression and overall combined agitation	Pearson
Medeiros et al.	2010	Multiple*	Various		75.7	NR	128	NR	128	probable AD	Mild, moderate, severe	MMSE	Mild: 23.2 Moderate: 16 Severe: 5.7	Mild: 8.41 Moderate: 9.61 Severe: 16.81	NR	CMAI and NPI	0.4	agitation	Pearson
Deslauriers et al.	2001	Canada	Care units from CHSLD		77.6	52.53%	99	NR	51.48	NR	NR	French version of FAST	4.3	1.9	nurse	IACM and IRPCM	0.74	agitation	Pearson
Gormley et al.	1998	United Kingdom	Old Age Psychiatry Service		78	64.29%	70	64.29%	70	Probable AD	Mild, moderate, severe	MMSE	12.8	62.41	Researcher	BEHAVE-AD and RAGE	0.81	Aggression	Spearman’s
Griffiths et al.	2020	NR	NR		85	73.80%	726	NR	691	NR	Mild, moderate, severe	FAST	>/= 4	NR	Researcher	CMAI/CMAI-O CMAI-O/ PAS CMAI-O/ NPI	CMAI & CMAI-O: 0.44 NPI & CMAI-O: 0.38 PAS & CMAI-O (AM): 0.583 PAS & CMAI-O (PM): 0.638	Agitation	Pearson
Kim et al.	2016	NR	NR		78.9	71.43%	63	71.43%	63	AD, VD, Lewy body	NR	MMSE	15.61	39.0625	Interviewer	KNPI-Q and K-NPI	0.52	Agitation/aggression	Spearman’s
Lam et al.	2006	China	Care and attention homes		82.04	58.40%	125	58.40%	125	AD, VD	severe	CMMSE	8.62	36.48	NR	CNPI and CCBS (incidence)	0.417	agitation	Spearman’s
Lam et al.	2001	China	Clinic		80.6	NR	71	NR	71	probable AD	NR	CMMSE	10.3	33.64	NR	C-BEHAVE-AD & Behavioural Symptoms Checklist	0.68	Aggression	Spearman’s
Logsdon et al.	1999	USA	21 sites		74.8	45.00%	148	45%	148	AD	NR	MMSE	13	56.25	NR	ABID and CMAI	0.62	Agitation	Pearson
Miller et al.	1995	Australia	Nursing Homes		80.4	68.50%	2445	NR	NR	NR	NR	MMSE	8.4	94.09	NR	1) CMAI to BEAM-D 2) CMAI to NHBPS	1) 0.91 2) 0.89 (day and evening); 0.64 (night)	Agitation	Pearson
Mungas et al.	1989	NR	Nursing facility		80.7	68.75%	16	68.75%	16	NR	NR	NR	NR	NR	Two authors	DBRS and Nurse’s Assessment rating scale	Severity: Physical: 0.69 Verbal: 0.38 Agitation: 0.73 Distress: Physical: 0.82 Verbal: 0.65 Agitation: 0.51	aggression and agitation	Pearson
Politis et al.	2004	Greece	Neuropsychiatry Clinic		71	40.00%	29	40%	29	NR	NR	MMSE	12.4	36	physician	H-NPI and EDS	0.57	aggression	Pearson
Roen et al.	2015	Norway	Nursing homes		84.9	69.20%	169	69.20%	169	NR	NR	MMSE	14	36	Registered Nurse	QUALID and NPI	0.497	agitation	NR
Cankurtaran et al.	2015	Turkey	Clinic		76.4	71.20%	125	71.20%	125	AD	NR	MMSE	16.6	40.96	independent rater	BEHAVE-AD/ NPI-C	0.52	Agitation/ Aggression	Pearson
Selbaek et al.	2007	Norway	Nursing homes		84.4	64.00%	50	NR	39	AD, VD, other	NR	MMSE	14.3	82.81	Geriatric psychiatrist	BEHAVE-AD and NPI	0.59	agitation/ Aggression	Spearman’s
Suh G-H	2004	Korea	Nursing home		79.6	82.50%	257	82.50%	257	AD, VD	NR	MMSE-K	13.9	34.81	Two psychologists	CMAI-K and BEHAVE-AD-K	0.81	agitation	Spearman’s
Victoroff et al.	1997	USA	University		75.6	67.83%	258	75.60%	258	AD, VD, mixed, other	NR	MMSE	NR	NR	NR	1) CMAI vs. CDBQ Agitation Subscale 2) BEHAVE-AD vs. CDBQ Agitation Subscale	1) 0.60 2) 0.59	Agitation	Pearson
Villanueva et al.	2003	USA	Residential Care Facilities		81.3	80.00%	40	80%	40	NR	Moderate to severe	GDS	5.28	0.6084	NR	PADE and CMAI	PADE : Part 1: 0.213; Part 2: 0.396; Part 3: 0.125	Aggression	NR
Weiner et al.	1998	NR	NR		72.3	60.70%	242	NR	206	probable AD	Mild to moderate	MMSE	13.38	62.57	NR	CMAI and BRSD	0.76	Aggression	Pearson
Weiner et al.	1997	USA	Clinic		73.6	58.00%	33	58%	33	probable AD	Mild to moderate	MMSE	16.5	31.36	NR	CMAI and CBRSD	0.397	Verbal agitation	NR
Youn et al.	2008	Korea	University and clinic		72.7	63.80%	268	63.80%	268	AD/ non-AD dementia	mild to moderate	MMSE	14.8	34.81	NR	K-NPI and BRSD-K	0.647	BRSD-K Irritability/aggression with NPI agitation	Pearson
Yodofsky et al.	1997	USA	Clinic		73.0	57.00%	39	NR	11	NR	NR	NR	NR	NR	NR	OASS and PAS	0.81	agitation	Pearson
Abe et al.	2015	Japan	dementia caregiver society		78.6	NR	NR	NR	792	AD	NR	MMSE	19	34.81	NR	ABSS and NPI	0.716	agitation	NR
Hurley et al.	1999	New England	LTC facilities	82.7		77.20%	57	77.20%	57	AD	NR	MMSE	5.2	23.04	NR	SOAPD and Agit-VAS	Physical: 0.75 Verbal: 0.85 Total: 0.90	agitation	Pearson
Smart et al.	2011	Canada	Long-term care facilities	85.4		0%	26	0%	26	AD	moderate to severe	MMSE	8.04	43.82	trained research assistant	1) ABS and CMAI 2) ABS and NPI-Agitation/aggression	1) 0.54 2) 0.10	aggressiveness/agitation	Spearman’s
Adama et al.	2013	France	Clinic	83.3		63.29%	79	NR	36	NR	Severe	MMSE	17.9	50.41	Trained researcher	CMAI and F-RAGE	0.73	aggressive behavior	Pearson
Curyto et al.	2021	USA	Community Living Centres	78.6		6.60%	302	NR	302	Ad, VD, Parkinson’s and Lewy Body, Other	NR	NR	NR	NR	NR	Agitated Reactive Behavior Scale and CMAI	0.53	Aggressive and agitated behaviour	Convergent correlation
Sun et al.	2022	Taiwan	Community daycare centres for older adults	79.9		62.3%	257	NR	NR	NR	NR	MMSE	10.28	54.5	Daycare nurse	1) C-CMAI-SF and C-NPI, 2) C-CMAI-SF and CSDD	1) Frequency: 0.58 Intensity: 0.48 Frequency x Intensity: 0.20 2) 0.56	Agitation	Pearson
Kratzer et al.	2023	Germany	Shared housing arrangements	82.8		76.2%	341	NR	254	NR	Mild-to-moderate	MMSE	NR	NR	Trained nursing staff	(1) CMAI-SF and NPI-NH “agitation” item, (2) CMI-SF and NPI-NH “agitation and restless behaviour” item	1) 0.66 2) 0.82	Agitation	Pearson

**†NR = Not Reported**

*Argentina, Brazil, Canada, France, Greece, Hungary, Italy, United States of America

**CMAI: Cohen-Mansfield Agitation Inventory; C-CMAI-SF: Chinese Cohen-Mansfield Agitation Inventory (Short-Form); CSDD: Cornell Scale for Depression in Dementia; RAS-PABS: Ryden-Aggression Scale – Physically Aggressive Behaviour Subscale; BEHAVE-AD: Behavioural Pathology in Alzheimer’s Disease Rating Scale; ABMI: Agitated Behaviours Mapping Instrument; NPI: Neuropsychiatric Inventory; C-NPI: Chinese-NPI; IACM (French version of CMAI); IRPCM (disruptive behaviour factor on the Revised Memory and Behavior Problems Checklist); RAGE: Rating Scale for Aggressive Behaviour in the Elderly; PAS: Pittsburgh Agitation Scale; CCBS: Chinese Version of the Challenging Behaviour Scale; ABID: Agitated Behaviour in Dementia Scale; NHBPS: Nursing Home Behaviour Problem Scale; DBRS: Disruptive Behaviour Rating Scale; EDS: Emotional Distress Scale; QUALID: Quality of Life in Late-Stage Dementia; CDBQ: California Dementia Behaviour Questionnaire; PADE: Pain Assessment for the Dementing Elderly; BRSD: Behavioural Rating Scale for Dementia; CBRSD: CERAD Behavioural Rating Scale for Dementia; OASS: Overt Agitation Severity Scale; ABSS: Abe’s BPSD Score Scale; SOAPD: Scale for Observation of Agitation in Persons with DAT (dementia of the Alzheimer’s type); Agit-VAS: Agitation-Visual Analogue Scale; ABS: Aggressive Behaviour Scale

Thirty articles comparing agitation and/or aggression tools (i.e., no reference standard), were included as part of our secondary objective [16, 22, 25, 31–58]. These studies determined the correlation between known agitation and/or aggression tools in PLWD. They were conducted in North America (*n* = 11) [25, 31, 33–35, 40, 48, 49, 51, 55, 57], Asia (*n* = 7) [32, 38, 39, 47, 52, 53, 56] South America (*n* = 1) [34], Europe (*n* = 8) [22, 34, 36, 43–46, 54, 58], and Australia (*n* = 1) [41]. Furthermore, four studies did not report their location [16, 37, 42, 50]. The studies were published between 1989 and 2023 [42, 58]. Dementia severity was determined mainly with the MMSE, or variations thereof, (*n* = 25) [22, 25, 31–34, 36–41, 43–48, 50–56, 58] with other studies using the Functional Assessment Staging Scale (FAST) (*n* = 2) [16, 35], and Global Deterioration Scale (GDS) (*n* = 1) [49]. Two study did not report how dementia severity was measured [42, 57]. The types of dementia reported include Alzheimer’s Disease, Vascular, Lewy Body, or general dementia not otherwise specified. However, multiple articles did not report the type (*n* = 11) [16, 22, 25, 35, 41–44, 49, 56, 58] or severity (*n* = 19) [25, 32, 33, 35, 37, 39–48, 53, 54, 56, 57] of dementia in their population.

Specific comparisons are listed in Table 4 and descriptions of each tool are shown in Supplemental Appendix 7.

### Outcomes of studies comparing between tools (table 4)

Pearson or Spearman’s correlation coefficients were reported among 28 articles, with 1 article not reporting the type of correlation coefficient [53] and another reporting the use of a non-specific convergent correlation coefficient [57].

#### CMAI

The CMAI was compared in 18 studies, demonstrating the highest correlation coefficient with the BEAM-D, with a Pearson’s value of 0.91 for agitation assessment [41]. The lowest correlation coefficient was a Pearson’s value of 0.20 between the CMAI and the ABMI in terms of overall combined agitation [33].

#### NPI

The NPI, or its various language translations, were compared to tools in (*n* = 11) studies. Among all tools, the K-NPI demonstrated the highest correlation with the ABSS, with a Correlation Coefficient value of 0.72 [52]. The type of correlation coefficient was not reported (Table 4) [52]. The weakest correlation was with the ABS, with a Spearman’s Correlation Coefficient of 0.10 [55].

#### BEHAVE-AD

The BEHAVE-AD, or variations of it, was compared to tools in (*n* = 7) studies. The highest correlation coefficient reported was a Spearman’s Correlation Coefficient of 0.81 between the BEHAVE-AD and RAGE, and between the CMAI-K and BEHAVE-AD-K [36, 47]. The lowest was a Pearson’s Correlation Coefficient of 0.52 between the BEHAVE-AD and the NPI-C [45].

DBRS: The DBRS was compared with only the Nurse’s Assessment Rating Scale in one study [42]. A series of Pearson’s correlation coefficients were reported for the severity and distress of physical and verbal aggression, as well as for physical and verbal agitation (Table 4).

PAS: The PAS was compared with the CMAI-O (*n* = 1), and the OASS (*n* = 1) in two studies [16, 25]. The highest correlation coefficient reported was with the OASS, with a Pearson’s correlation coefficient of 0.81 [25].

SOAPD: The SOAPD scale was only compared to the Agit-VAS scale (*n* = 1) [54]. The total (verbal and physical) Pearson correlation coefficient score for agitation was 0.90.

### Risk of bias assessment

#### Studies comparing tools to a reference standard: (supplemental appendix 3)

Included studies demonstrated low risk that the included patients and target condition did not match the review question (*n* = 6) [14, 15, 21–24]. Two studies reported blinding between the index and reference tools, and had low concern that the conduct of the index test was biased [22, 24]. Another three studies had unclear blinding between index and reference tools, potentially introducing bias in the results [14, 21, 23]. One study reported no blinding [15]. Lastly, there was concern about the time between administration of the reference standard and index tool across studies (*n* = 6) [14, 15, 21–24].

#### Studies comparing tools: (supplemental appendices 4 and 5)

Most included studies demonstrated low concern that the included patients did not match the review question (*n* = 29) [22, 31–36, 16, 37–54, 25, 58, 56, 57], with one study demonstrating unclear concern [55] due to unclear exclusion criteria. Many studies did not indicate whether test administrators were blinded (*n* = 22) [25, 33, 34, 36, 38–42, 44, 45, 47–54, 56–58], with (*n* = 3) [16, 32, 55] studies indicating no blinding, thus there was varying concern regarding the conduct between the two tools (Supplemental Appendices 4 and 5). Nonetheless, there was low concern that the target condition (i.e., agitation and/or aggression) as defined by both tools did not match the review question across studies (*n* = 30) [16, 22, 25, 31–36, 36–58]. Additionally, the time interval between administration of both agitation and/or aggression tools was often not reported or ambiguous (*n* = 29) [16, 22, 25, 31–34, 36–58]. This area could have also introduced bias in the results, where knowledge about the first tool could have influenced participants’ responses on the second tool.

## Discussion

We identified six studies comparing either agitation or aggression tools to reference standards. To detect the presence of agitation, the agitation domain of the Spanish NPI demonstrated the highest sensitivity of 100% [23] compared to the agitation subsection of the Spanish CAMDEX, in a single study. In comparison, the NBRS, and PAS demonstrated similarly high sensitivities of 95.2% and 85.7%, respectively, both compared to a psychiatrist’s diagnosis of agitation and/or aggression [24]. The Spanish NPI has a higher sensitivity compared to its English counterpart, likely due to differences in study design, along with the use of the CAMDEX as the reference standard compared to other studies [23]. Overall, based on single studies, the Spanish NPI, NBRS, and PAS appear favorable among PLWD to detect agitation.

Mauleon et al. (2020) mapped items from the CMAI and NPI-Clinician (NPI-C) onto IPA agitation criteria domains to create IPA-informed agitation scales [15]. Both the NPI-C-IPA and the NPI-C demonstrated reasonable MCID scores (-5 and − 3, respectively) [15]. Their results suggest how the IPA agitation domain may be helpful to improve the agitation diagnostic abilities of a tool, compared to those that do not involve the IPA (i.e. NPI-C and CMAI).

From our analysis, only one study reported diagnostic accuracy measures for an assessment tool assessing aggression (i.e. F-RAGE) [22]. In the literature, there is a lot of overlap and mixing between agitation and aggression among studies [59]. This issue makes it difficult to identify validity constructs for each separate symptom. More research is thus needed to validate aggression tools to understand their efficacy at bedside.

Another 30 studies were identified that compared the correlation in agitation or aggression symptoms between two or more tools. Correlation coefficients were most commonly drawn between the CMAI and other agitation tools, in 18 studies. The highest correlation coefficient drawn was between the CMAI and BEAM-D of 0.91 [41]. Although useful to understand the comparative validity of these tools, clinically this can be harder to use when it comes to implementation and accuracy at bedside.

Due to widespread disagreement on the definition of agitation before 2015, the best reference standard prior was considered a physician’s clinical diagnosis, as there were no set criteria for agitation among PLWD [14, 60]. Without a reference standard diagnosis, the validity of older tools lacks clarity, with most studies conducted prior to 2015 examining construct validity rather than diagnostic accuracy measures (e.g. sensitivity, specificity). We have found seven tools compared to a reference standard such as clinician diagnosis, but still few studies use the IPA criteria.

Currently the most commonly used agitation and/or aggression scales among RCTs include the BEHAVE-AD (*n* = 10), the agitation/aggression domain of the NPI (along with variations of it) (*n* = 39), and the CMAI (*n* = 173) [12]. However, we only found (*n* = 1) and (*n* = 2) studies validating the BEHAVE-AD and NPI, respectively, compared to a reference standard [21, 23]. No diagnostic accuracy studies reporting sensitivity or specificity measures were obtained for the CMAI. Therefore, the validity of these tools are unclear, despite their recurrent use in clinical trials. More research is thus needed to validate the most common agitation and/or aggression tools amongst PLWD to improve clinical research. Additionally we found no evidence on tools such as Behaviour and Symptom Mapping Tools and the Aggressive Behaviour Scale in the RAI-Minimum Data Set (MDS) 2.0 [59]. The Behaviour and Symptom Mapping Tools primarily notes behavioural trends in response to events, in a qualitative fashion, and are often a key part of assessing antecedent events for behaviors [61], so it is unlikely tools such as this may be compared to a reference standard.

Despite the myriad of tools, few studies have assessed them for diagnostic accuracy. Future studies can address gaps looking at comparisons of diagnostic accuracy measures between the many tools, different languages, or ethnicities, various pathologies and severity of dementia, as well as different types of care settings. The CMAI, and BEHAVE-AD are commonly used scales in the literature, but more is needed to examine diagnostic accuracy of these tools. Certain tools as demonstrated by Mauleon et al. (2021) and Sano et al. (2022) overlap with the IPA criteria of agitation, more is needed to compare to the IPA criteria [14, 15].

### Strengths and limitations

Our study had a rigorous search procedure and following all PRISMA reporting guidelines. Although we completed an extensive search, few studies with diagnostic accuracy measurements were identified, thus a meta-analysis could not be performed. As well, separate searches for the found instruments were not performed after relevant articles were included, thus serving as a potential limitation to our data collection methods. We also did not include the names of specific tools in our searches. There is also the chance that we may have missed literature despite the exhaustive nature of our search. We did not have any language restrictions on studies, however the use of translation software (i.e. Google translate) may have posed as a limitation to the interpretation of results.

Among included studies, the risk of bias assessment showed that many (*n* = 24) did not indicate whether administrators were blinded to one another, or did not specify the flow and timing of the study (*n* = 30). These unclear aspects can impact the precision in determining a given test’s diagnostic accuracy. Additionally, given the limited number of included studies, we lack data on the accuracy of these tools across different dementia pathologies, dwellings (community vs. long term care) or severities of dementia.

## Conclusion

We found few studies reporting a comparison of agitation and/or aggression tools to a reference standard. Thus, we lack evidence on the sensitivity and specificity of these tools. From our current knowledge, the agitation domain of the Spanish NPI, NBRS, and PAS demonstrated the highest sensitivity for assessing symptoms of agitation and/or aggression, yet their accuracy at bedside is still unclear. More rigorous studies are needed to understand the diagnostic accuracy of tools for the detection agitation or aggression in PLWD.

### Electronic supplementary material

Below is the link to the electronic supplementary material.

Supplementary Material 1

## Data Availability

The datasets used and/or analyzed during the current study are available from the corresponding author on reasonable request.
