# Supplementary material for: Detecting agitation and aggression in persons living with dementia: a systematic review of diagnostic accuracy
Source: BMC Geriatr. 2024 Jun 26;24:559. doi: 10.1186/s12877-024-05143-6 (PMC11210082; doi:10.1186/s12877-024-05143-6)
Supplement: Supplementary file 1 — Supplementary Material 1 [file 12877_2024_5143_MOESM1_ESM.docx]

**Supplemental Appendix:**

**Appendix 1: The Search Strategy Terms Used in the Databases MEDLINE, EMbase and PsycINFO from inception until August 14 2021.**

|  | **Diagnostic Accuracy Terms** | **Agitation and/or Aggression Terms** | **Dementia Terms** |
| --- | --- | --- | --- |
| **Keywords** | Valid*  Screen*  Tool*  Questionnaire*  Diagnostic Accuracy*  Psychometric*  Survey*  Test*  Instrument*  Inventory*  Sensitivity*  Specificity*  Neuropsychological Tests* | Aggress*  Agitat* | Parkinson*s Disease adj10 Dementia  Alzheimer*  Lewy Bod*  Neurocognitive*  Frontotemporal*  Dementia* |
| **MEDLINE Terms** | Screening  Mass Screening  ROC Curve  Sensitivity and Specificity  Questionnaires  Psychometrics  Neuropsychological Test  Clinical Decision Making | Aggression  Psychomotor Agitation | Lewy Body  Dementia  Frontotemporal Dementia  Dementia, Vascular  Dementia, Multi-Infarct  Alzheimer’s Disease  Parkinson’s Disease |
| **EMbase Terms** | Screening  Mass Screening  Screening Test  Receiver Operating Characteristic  “Sensitivity and Specificity”  Diagnostic Accuracy  Questionnaire  “Tool Use”  Psychometry  Neuropsychological Tests  Validity  Clinical Decision Making | Aggression  Cohen-Mansfield Agitation Inventory  Agitation | Lewy body  Multi-infarct Dementia  Pick Presenile Dementia  Frontal variant frontotemporal dementia  Semantic dementia  Presenile dementia  Senile dementia  Frontotemporal dementia  Dementia  "mixed depression and dementia"  Alzheimer’s Disease  Parkinson’s Disease |
| **PsycINFO Terms** | Screening  Mass Screening  Screening Test  Questionnaire  Tool Use  Psychometrics  Neuropsychological Assessment  Test Validity | Aggressive Behaviour  Agitation | Lewy Body  Presenile Dementia  Dementia  Senile Dementia  Semantic Dementia  Dementia with Lewy Bodies  Vascular Dementia  Alzheimer’s Disease  Parkinson’s Disease |

**Appendix 2: The grey literature databases searched to further exhaust the search strategy**

| Mental Health Organizations | - CADTH - CCSMH - CMHA - Mental Health Canada - MHE-SME - Mental Health America - CAGP - EAGP - AAGP |
| --- | --- |
| Cognitive Sites | - Alzheimer.ca - Alzheimer-europe.org/ - Alz.org - Alzfdn.org/ |
| General Grey Databases | - Government of Canada - GreyNet International - SIGLE (System for Information on Grey Literature in Europe) |
| Search Engines | - TRIP database - Google Scholar |
| International | - Agency for Healthcare Research and Quality - World Health Organization |
| Thesis | - Centre for Resarch Libraries Foreign Dissertation - DART-Europe E-theses Portal - Electronic Theses Online Service (ETHOS) \| British Library - Thesis Canada Portal |

**Appendix 3: The Risk of Bias for Studies that Compared Tools to a Reference Standard:**

|  |  | **Ismail 2013** | **Grey. Adama 2013** | **Vilalta-Franch 1999** | **Rosen et al., 2006** | **Mauleon et al., 2020** | **Sano et al. 2022** |
| --- | --- | --- | --- | --- | --- | --- | --- |
| **Participant Selection** | **Was a consecutive or random sample of participants enrolled? (Yes/No/Unclear)** | Yes | Unclear | Unclear | Unclear | Yes | Unclear |
|  | **Was a case-control design avoided? (Yes/No/Unclear)** | Yes | Yes | Yes | Yes | Yes | Yes |
|  | **Did the study avoid inappropriate exclusions? (Yes/No/Unclear)** | Yes | Yes | Yes | Yes | Yes | Yes |
|  | **Could the selection of participants have introduced bias? (Low/High/Unclear)** | Low | Unclear | Unclear | Unclear | Low | Unclear |
|  | **Is there concern that the included patients do not match the review question? (Low/High/Unclear)** | Low | Low | Low | Low | Low | Low |
| **Index Test** | **Were the index test results interpreted without knowledge of the results of the reference standard? (Yes/No/Unclear)** | Unclear | Yes | Unclear | Yes | No | Unclear |
|  | **If a threshold was used, was it pre-specified? (Yes/No/Unclear)** | No | No | No | No | No | No |
|  | **Could the conduct or interpretation of the index test have introduced bias? (Low/High/Unclear)** | Unclear | Low | Unclear | Low | Unclear | Unclear |
|  | **Is there concern that the index test, its conduct or interpretation differ from the review question? (Low/High/Unclear)** | Unclear | Low | Unclear | Low | Unclear | Unclear |
| **Reference Standard** | **Is the reference standard likely to correctly classify the target condition? (Yes/No/Unclear)** | Yes | Yes | Yes | Yes | Yes | Yes |
|  | **Were the reference standard results interpreted without knowledge of the results of the index test? (Yes/No/Unclear)** | Unclear | Yes | Unclear | Yes | No | Unclear |
|  | **Could the reference standard, its conduct, or its interpretation have introduced bias? (Low/High/Unclear)** | Unclear | Low | Unclear | Low | Unclear | Unclear |
|  | **Is there concern that the target condition as defined by the reference standard does not match the review question? (Low/High/Unclear)** | Low | Low | Low | Low | Low | Low |
| **Flow and Timing** | **Was there an appropriate time interval between index test and reference standard? (Yes/No/Unclear)** | Unclear | Unclear | Unclear | Unclear | Unclear | Unclear |
|  | **Did all patients receive a reference standard? (Yes/No/Unclear)** | Yes | Yes | Yes | Yes | Yes | Yes |
|  | **Did all patients receive the same reference standard? (Yes/No/Unclear)** | Yes | Yes | Yes | Yes | Yes | Yes |
|  | **Were all patients included in the analysis? (Yes/No/Unclear)** | Yes | Yes | Unclear | Yes | Yes | Yes |
|  | **Could the patient flow have introduced bias? (Low/High/Unclear)** | Unclear | Unclear | Unclear | Unclear | Unclear | Unclear |

**Appendix 4: The Risk of Bias Assessment for Studies Comparing Tools (Part 1):**

|  |  | **Whall 2013** | **Choy, 2001** | **Cohen-Mansfield 2004** | **Medeiros 2010** | **Landreville et al. 2001** | **Gormley 1998** | **Griffiths 2019** | **Kim 2016** | **Lam 2006** | **Lam 2001** | **Logsdon 1999** | **Miller 1995** | **Mungas 1989** |
| --- | --- | --- | --- | --- | --- | --- | --- | --- | --- | --- | --- | --- | --- | --- |
| **Participant Selection** | **Was a consecutive or random sample of participants enrolled? (Yes/No/Unclear)** | Yes | Unclear | Unclear | Unclear | Yes | Unclear | Unclear | Unclear | Unclear | Unclear | Unclear | Unclear | Unclear |
|  | **Was a case-control design avoided? (Yes/No/Unclear)** | Yes | Yes | Yes | Yes | Yes | Yes | Yes | Yes | Yes | Yes | Yes | Yes | Yes |
|  | **Did the study avoid inappropriate exclusions? (Yes/No/Unclear)** | Yes | Yes | Yes | Yes | Yes | Yes | Yes | Yes | Yes | Yes | Yes | Yes | Yes |
|  | **Could the selection of participants have introduced bias? (Low/High/Unclear)** | Low | Unclear | Unclear | Unclear | Low | Unclear | Unclear | Unclear | Unclear | Unclear | Unclear | Unclear | Unclear |
|  | **Is there concern that the included patients do not match the review question? (Low/High/Unclear)** | Low | Low | Low | Low | Low | Low | Low | Low | Low | Low | Low | Low | Low |
| **Index Test** | **Were the index test results interpreted without knowledge of the results of the reference standard? (Yes/No/Unclear)*** | CMAI: Yes | CMAI: No | CMAI: Unclear | CMAI: Unclear | CMAI: Unclear | RAGE: Unclear | CMAI: No | KNPI: Yes | CNPI: Unclear | C-BEHAVE:  Unclear | ABID: Yes | CMAI:  Unclear | DBRS: Unclear |
|  | **If a threshold was used, was it pre-specified? (Yes/No/Unclear)** | No | No | No | No | No | No | No | No | No | No | No | No | No |
|  | **Could the conduct or interpretation of the index test have introduced bias? (Low/High/Unclear)** | Low | High | Unclear | Unclear | Unclear | Unclear | Unclear | Low | Unclear | Unclear | Low | Unclear | Unclear |
|  | **Is there concern that the index test, its conduct or interpretation differ from the review question? (Low/High/Unclear)** | Low | High | Unclear | Unclear | Unclear | Unclear | Unclear | Low | Unclear | Unclear | Unclear | Unclear | Unclear |
| **Reference Standard** | **Is the reference standard likely to correctly classify the target condition? (Yes/No/Unclear)**** | Ryden: Yes | C-BEHAVE: Yes | ABMI: Yes | NPI: Yes | Nurse  Assessment: Yes | BEHAVE-AD: Yes | PAS: Yes | NPI: Yes | CCBS: Yes | Behaviour Checklist: Yes | CMAI: Yes | BEAM-D: Yes | Nurse's  Assessment: Yes |
|  | **Were the reference standard results interpreted without knowledge of the results of the index test? (Yes/No/Unclear)** | Yes | No | Unclear | Unclear | Unclear | Unclear | No | Yes | Unclear | Unclear | Unclear | Unclear | Unclear |
|  | **Could the reference standard, its conduct, or its interpretation have introduced bias? (Low/High/Unclear)** | Low | Unclear | Unclear | Unclear | Unclear | Unclear | Unclear | Low | Unclear | Unclear | Unclear | Unclear | Unclear |
|  | **Is there concern that the target condition as defined by the reference standard does not match the review question? (Low/High/Unclear)** | Low | Low | Low | Low | Low | Low | Low | Low | Low | Low | Low | Low | Low |
| **Flow and Timing** | **Was there an appropriate time interval between index test and reference standard? (Yes/No/Unclear)** | Unclear | Unclear | Unclear | Unclear | Yes | Unclear | Unclear | No | Unclear | Unclear | Unclear | Unclear | Unclear |
|  | **Did all patients receive a reference standard? (Yes/No/Unclear)** | Yes | Yes | Yes | Yes | Yes | Yes | Unclear | Yes | Unclear | Yes | Yes | Yes | Yes |
|  | **Did all patients receive the same reference standard? (Yes/No/Unclear)** | Unclear | Yes | Yes | Yes | Yes | Yes | Yes | Yes | Yes | Yes | Yes | Yes | Yes |
|  | **Were all patients included in the analysis? (Yes/No/Unclear)** | Yes | Yes | Yes | Yes | Yes | Yes | Yes | Yes | Yes | Yes | Yes | No | Yes |
|  | **Could the patient flow have introduced bias? (Low/High/Unclear)** | Unclear | Unclear | Unclear | Unclear | Low | Unclear | Unclear | Unclear | Unclear | Unclear | Unclear | Unclear | Unclear |

*Interpreted as whether one of the tools measured for correlation was interpreted without knowledge of the second tool. The tool specified is listed

**Interpreted as whether the second tool measured for correlation is likely to classify the target condition. The tool specified is listed.

**Appendix 5: The Risk of Bias Assessment for Studies Comparing Tools (Part 2):**

|  |  | **Politis 2004** | **Roen 2015** | **Cankurtaran 2015** | **Selboek 2007** | **Suh 2004** | **Victoroff 1997** | **Villaneuva 2003** | **Weiner 1998** | **Weiner 1997** | **Youn 2008** | **Yodofky 1997** | **Abe 2015** | **Hurley 1999** | **Smart 2011** | **Kratzer 2023** | **Sun, 2022** | **Curyto 2021** |
| --- | --- | --- | --- | --- | --- | --- | --- | --- | --- | --- | --- | --- | --- | --- | --- | --- | --- | --- |
| **Participant Selection** | **Was a consecutive or random sample of participants enrolled? (Yes/No/Unclear)** | Yes | Unclear | Unclear | Unclear | Unclear | Yes | Unclear | Unclear | Yes | Unclear | Yes | Unclear | Unclear | Unclear | Unclear | Unclear | Unclear |
|  | **Was a case-control design avoided? (Yes/No/Unclear)** | Yes | Yes | Yes | Yes | Yes | Yes | Yes | Yes | Yes | Yes | Yes | Yes | Yes | Yes | Yes | Yes | Yes |
|  | **Did the study avoid inappropriate exclusions? (Yes/No/Unclear)** | Yes | Yes | Yes | Yes | Yes | Yes | Yes | Yes | Yes | Yes | Yes | Yes | Yes | No | Yes | Unclear | Yes |
|  | **Could the selection of participants have introduced bias? (Low/High/Unclear)** | Low | Unclear | Unclear | Unclear | Unclear | Low | Unclear | Unclear | Low | Unclear | Low | Unclear | Unclear | Unclear | Unclear | Unclear | Unclear |
|  | **Is there concern that the included patients do not match the review question? (Low/High/Unclear)** | Low | Low | Low | Low | Low | Low | Low | Low | Low | Low | Low | Low | Low | Unclear | Low | Low | Low |
| **Index Test [Test 1]** | **Were the index test [test 1] results interpreted without knowledge of the results of the reference standard? (Yes/No/Unclear)*** | HNPI: Yes | NPI: Unclear | NPI-C: Unclear | NPI: Yes | CMAI: Unclear | CMAI: Unclear | CMAI: Unclear | CMAI: Unclear | CMAI: Unclear | BRSD-K: Unclear | OASS: Unclear | NPI: Unclear | SOAPD: Unclear | NPI: No | CMAI-SF: Unclear | C-CMAI-SF: Unclear | ARBS: Unclear |
|  | **If a threshold was used, was it pre-specified? (Yes/No/Unclear)** | No | No | No | No | No | No | No | No | No | No | No | No | No | Yes | No | No | No |
|  | **Could the conduct or interpretation of the index test have introduced bias? (Low/High/Unclear)** | Low | Unclear | Unclear | Low | Unclear | Unclear | Unclear | Unclear | Unclear | Unclear | Unclear | Unclear | Unclear | High | Unclear | Unclear | Unclear |
|  | **Is there concern that the index test, its conduct or interpretation differ from the review question? (Low/High/Unclear)** | Low | Unclear | Unclear | Low | Unclear | Unclear | Unclear | Unclear | Unclear | Unclear | Unclear | Unclear | Unclear | High | Unclear | Unclear | Unclear |
| **Reference Standard [Test 2]** | **Is the reference standard [Test 2] likely to correctly classify the target condition? (Yes/No/Unclear)**** | BPRS: Yes | QUALID: Yes | BEHAVE-AD: Yes | BEHAVE-AD:  Yes | BEHAVE-AD:  Yes | CDBQ: Yes | PADE: Yes | BRSD: Yes | CBRSD: Yes | NPI-K: Yes | PAS: Yes | ABS: Yes | Agit-VAS: Yes | CMAI/ABS:  Yes | NPI-NH: Yes | NPI: Yes | CMAI-SF: Yes |
|  | **Were the reference standard results interpreted without knowledge of the results of the index test? (Yes/No/Unclear)** | Yes | Unclear | Unclear | Yes | Unclear | Unclear | Unclear | Unclear | Unclear | Unclear | Unclear | Unclear | Unclear | No | Unclear | Unclear | Unclear |
|  | **Could the reference standard, its conduct, or its interpretation have introduced bias? (Low/High/Unclear)** | Low | Unclear | Unclear | Low | Unclear | Unclear | Unclear | Unclear | Unclear | Unclear | Unclear | Unclear | Unclear | Unclear | Unclear | Unclear | Unclear |
|  | **Is there concern that the target condition as defined by the reference standard does not match the review question? (Low/High/Unclear)** | Low | Low | Low | Low | Low | Low | Low | Low | Low | Low | Low | Low | Low | Low | Low | Low | Low |
| **Flow and Timing** | **Was there an appropriate time interval between index test and reference standard? (Yes/No/Unclear)** | Unclear | Unclear | Unclear | Unclear | Unclear | Unclear | Unclear | Unclear | No | Unclear | Unclear | Unclear | Unclear | Unclear | Unclear | Unclear | Unclear |
|  | **Did all patients receive a reference standard? (Yes/No/Unclear)** | Yes | Yes | Yes | Yes | Yes | Yes | Yes | Yes | Yes | Yes | Yes | Yes | Yes | Yes | Yes | Yes | Yes |
|  | **Did all patients receive the same reference standard? (Yes/No/Unclear)** | Yes | Yes | Yes | Yes | Yes | Yes | Yes | Yes | Yes | Yes | Yes | Yes | Yes | Yes | Yes | Yes | Yes |
|  | **Were all patients included in the analysis? (Yes/No/Unclear)** | Yes | Unclear | Yes | Yes | Yes | No | Yes | No | Yes | No | No | Yes | Yes | No | Yes | Yes | No |
|  | **Could the patient flow have introduced bias? (Low/High/Unclear)** | Unclear | Unclear | Unclear | Unclear | Unclear | Unclear | Unclear | Unclear | Unclear | Unclear | Unclear | Unclear | Unclear | Unclear | Unclear | Unclear | Unclear |

*Interpreted as whether one of the tools measured for correlation was interpreted without knowledge of the second tool. The tool specified is listed.

**Interpreted as whether the second tool measured for correlation is likely to classify the target condition. The tool specified is listed.

**Appendix 6: Descriptions of Each Tool Comparing to a Reference Standard.**

| **Name of Tool** | **Abbreviation** | **Versions of Tools Used Among Studies** | **Number of Items** | **Rater** | **Format** | **Domains Covered** | **Administration Time** | **Key Tool Features** |
| --- | --- | --- | --- | --- | --- | --- | --- | --- |
| Behavioral Pathology in Alzheimer’s Disease Rating Scale | BEHAVE-AD | E-BEHAVE-AD^21^ | BEHAVE-AD: 25^21^  E-BEHAVE-AD:12^21^ | Completed by a knowledgeable informant (ie. Caregiver, nurse, clinician)^21,28^ | Severity Scale^21^ | Delusions, hallucinations, affective disturbances, anxiety and phobias, aggressiveness, activity disturbances, sleep disturbances ^28,36^ | 20 minutes^63^ | - Used for dementia-related behavioural changes^28^ - Specifically used among patients with Alzheimer’s Disease^28^ - Contains a global assessment of overall magnitude of disturbance to the caregiver and/or danger to the patient caused by behavioural symptoms^39^ |
| Neuro-behavioural Rating Scale | NBRS | N/A | 27^21^ | Observer-rated^21^ | Observational and administered on a Likert Scale^21^ | aggression, agitation, hostility, delusions, hallucinations, and suspiciousness^21^ | NR | - Designed based on the Brief Psychiatric Rating Scale^21^ - Symptoms are rated from 0-6 with 0 indicating the absence of a symptom to 6 being an extremely severe symptom^21^ - Target symptoms are considered present at a score of ≥3 at baseline and resolved if the score decreased to ≤2^21^ |
| Neuropsychiatric Interview | NPI | Spanish-NPI^23^  NPI-C-IPA | 7-9 items within each of 12 domains^34^ | Knowledgeable Informant ^28^ | Questionnaire on Likert scale^16^ | 10 behavioural and 2 neurovegetative areas: (delusions, hallucinations, agitation and/or aggression, depression and/or dysphoria, anxiety, elation and/or euphoria, apathy and/or indifference, disinhibition, irritation and/or lability, aberrant motor behaviour, sleep and nighttime behaviour disorders, and appetite and/or eating disorders)^34^ | 10-20 minutes^34^ | - Consists of 12 domains assessing various neuropsychiatric disturbances^26^ - Common scale used among persons with dementia and other neurological disorders - Assesses the presence, frequency and severity of each behaviour in the previous month - Also assesses the level of caregiver distress due to each neuropsychiatric symptom^26^ - Only one domain of the NPI assesses agitated behaviour - A screening question is given for each of the 12 domains. If a disturbance is indicated in the screening question, the rest of the 6-8 sub-questions will be asked pertaining to that behaviour^26^ |
| Pittsburgh Agitation Scale | PAS | N/A | 4^25^ | Trained Researcher or clinical Staff^16^ | Observational Scale^25^ | Describes the severity of agitation in four behavioral domains: aberrant vocalizations, motor agitation, aggressiveness, and resisting care^25^ | Observations should be conducted between 1-8hrs^16^ | - The observer uses behavioral anchors to assign scores within each behavior group.^24^ - A higher score reflects higher agitation, with scores ranging from 0-4 for each category^24^ |
| Rating Scale for Aggressive Behavior in the Elderly | RAGE | F-RAGE^22^ | 21^22^ | Informant Rated (ie. caregiver, nurse), or administered by a trained researcher ^22^ | Likert-Scale^22^ | Verbal and Physical symptoms of aggression | 5-10 minutes^22^ | - Specifically developed for finding aggressive behaviour in institutionalized or hospitalized elderly patients^64^ |
| Cohen-Mansfield Agitation Inventory | CMAI | - CMAI-K (Korean version) ^47^ - CCMAI (Chinese version) ^32^ - CMAI-O (observational) ^16^ - IACM (French version) ^35^ - CMAI-IPA | 29^47^ | formal caregiver/informant^32^ | Observational on Likert scale or structured interview with frequency rating scale^31,33^ | physically aggressive, physically non-aggressive, verbally aggressive, verbally non-aggressive^65^ | CMAI: <30 minutes (Weiner et al., 1998)  CMAI-O: 5 hours^16^ | - Used extensively in nursing homes^40^ - Has widespread use in clinical trials of pharmacological and psychosocial interventions^40^ |

†**NR = Not Reported**

**Appendix 7: Descriptions of Each Tool Compared with Other Tools.**

| **Name of Tool** | **Abbreviation** | **Versions of Tools Used Among Studies** | **Number of Items** | **Rater** | **Format** | **Domains Covered** | **Administration Time** | **Key Tool Features** |
| --- | --- | --- | --- | --- | --- | --- | --- | --- |
| Agitated Behavior in Dementia scale | ABID | N/A | 16 | Completed by the caregiver | Questionnaire format | Aggression and Agitation | ~15 minutes | - Designed specifically to evaluate frequency of, and caregiver reaction to, agitated behaviors in community-residing dementia patients^40^ - Targets mild to moderate non-institutionalized AD patients^40^ |
| Agitated Behaviors Mapping Instrument | ABMI | N/A | 14 | Trained Research Assistants | Observational | Physical and Verbal agitation | Observed for 3min every 30min for 13hrs/day (8am-9pm) | - Has an item to measure disruptiveness of observed agitation - Takes into account temporal differences among patients^53^ |
| Abe's BPSD score scale | ABSS | N/A | 10 | Completed by the caregiver | Frequency and severity scale | Wandering, eating/toilet problem, delusion/hallucination, offensive/abusive words, day-night reversal, excitation/agitation, apathy/indifference, depressive mood, violent force, high irritability | ~1 minute | - A quick and simple test to assess BPSD in patients with mild to moderate dementia^53^ - Shorter than the NPI with less time needed to administer test^53^ |
| Aggressive Behavior Scale | ABS | N/A | 4 | Trained Observer^66^ | Frequency Scale/ Observational | Verbal and Physical Aggression | Administered over a period of 7 days^67^ | - Used to assess agitated and aggressive behaviour - Frequency of ABS items are coded as: not exhibited (0), occurred 1-3 days in the past 7 days (1), occurred 4-6 days in the past 7 days but less than daily (2), behaviour occurred daily (3) - Score for ABS ranges from -12, with higher scores indicating higher frequency of behaviours^67^ |
| Agitation- Visual Analogue Scale | Agit-VAS | N/A | NR | Trained Researcher | Observational | Overall Agitation | NR | - Uses a 100mm horizontal line with anchors of “none” to “extreme” to rate observed agitation - The research observer makes a global assessment of overall agitation^54^ |
| Behavioral and Emotional Activities Manifested in Dementia | BEAM-D | N/A | 10 | NR | Observational | Hostility, aggression, destruction, disruption, uncooperativeness, non-compliance, attention-seeking, sexually inappropriate behaviour, wandering, hoarding | NR | - Designed to assess behavioural disorders in cognitively impaired older people^41^ - Developed for the operational assessment of troublesome and disruptive behaviours among persons with dementia^68^ |
| Behavioural Symptom Checklist | N/A | N/A | 54^39^ | Caregiver^39^ | Checklist evaluating the frequency of behaviour^39^ | sleep and appetite disturbance, wandering, perceptual abnormalities, hallucinating experience, abnormal thought or ideas, odd behavior, repetitive actions, mood changes^39^ | NR | NR |
| CERAD Behavioral Rating Scale for Dementia | CBRSD | BRSD-K (Korean version) ^52^ | 48^50^ | Informant-Rated ^51^ | Questionnaire with likert scale/structured caregiver interview^51^ | depressive features, psychotic features, defective self-regulation, irritability/**agitation**, vegetative features, apathy, **aggression**, affective lability ^50^ | <30 minutes^50^ | NR |
| Chinese Version of the Challenging Behaviour Scale | CCBS | CBS | 25 | Trained or Untrained Formal Caregivers | NR | NR | NR | - Designed for use in residential care settings^38^ - Covers global and specific behaviours in residents with dementia^38^ |
| California Dementia Behaviour Questionnaire | CDBQ- Agitation Subscale | N/A | 87 | Caregivers^48^ | Questionnaire^48^ | Agitation Subscale^48^ | NR | - Primarily derived from frequency of symptoms^48^ |
| Disruptive Behaviour Rating Scale | DBRS | N/A | 21 | Independent Raters | Questionnaire with severity rating scales | Physical and verbal aggression, agitation, wandering, total disruptive behaviour | ~5-10 minutes^69^ | - A detailed scale assessing a narrower range of agitated and/or aggressive behaviour^42^ - Used among persons with dementia/ Alzheimer’s Disease - Assesses the frequency and severity of disruptive behaviour across all four dimensions^70^ |
| Emotional Distress Scale | EDS | N/A | 5 | Caregiver | Likert Scale | Fear, Psychic Anxiety, sleep disturbance, restrictions of daily living activities, fatigue | ~15 minutes | - Assesses distress in caregiving - Can be used among caregivers of dementia patients, of patients with severe cardiac failure undergoing heart transplantation, and parents of patients with schizophrenia in emergency outpatient setting^43^ |
| Revised Memory and Behaviour Problems Checklist | N/A | IRPCM (French Version) | 24 | Caregiver | Observational | Memory-related problems, depression, and disruptive behaviours (ie. verbal aggression) | NR | - Measures frequency of observable behavioural problems in dementia patients^71^ - Behaviours are rated from “never occurs” to “occurs daily or more often” on a scale from 1-4^71^ - Also evaluates the caregiver’s reaction to each behaviour, providing an index of the impact of each behaviour on the caregiver^71^ |
| Nursing Home Behaviour Problem Scale | NHBPS | N/A | 29 | Completed by Nursing Staff | Observational | Agitated and aggressive behavioural symptoms | 3-5 minutes | - The rater reports the frequency of each behaviour in the past 3 days, using a 5-point frequency of occurrence scale (0 = never to 4 = always)^72^ - A higher score indicates a greater level of behavioral problems^72^ |
| Overt Agitation Severity Scale | OASS | N/A | 16 | Nursing Staff | Observational | 47 observable characteristics of agitation subcategorized into 12 units^25^ | ~15 minutes | - Constructed to rate specifically agitation instead of a large range of behavioral problems^25^ - Eliminates inference and subjective clinical judgements^25^ |
| Pain Assessment for the Dementing Elderly | PADE | N/A | 24 | Caregiver | Likert Scale and Multiple Choice questionnaire | Physical features, global assessment and functional assessment | ~5-10 minutes | - Aids in distinguishing between pain and agitation^49^ |
| Psychogeriatric Dependency Rating Scale – Behavioural Subscale | PGDRS | N/A | NR | NR | NR | Physical and verbal aggression | NR | NR |
| Quality of Life in Late-Stage Dementia | QUALID | Swedish version, Spanish version, Norwegian version | 11 | Proxy-rated (ie. family member or healthcare worker)^44^ | Structured, proxy-based interview^44^ | Observable behaviours indicative of quality of life^44^ | NR | - Developed for those with late-stage dementia in nursing homes^44^ - Strong correlation between QUALID and depressive symptoms^44^ |
| Ryden-Aggression Scale – Physically Aggressive Behaviour Subscale | RAS-PABS | N/A | 17^31^ | NR | Observational^31^ | Physical aggression^31^ | ~1-8 hours | NR |
| Scale for Observation of Agitation in Persons with DAT (dementia of the Alzheimer’s type) | SOAPD | N/A | 7 | Research assistants and clinical staff | Observational | Agitated behaviours | ~5 minute observation periods | - Measures observed agitation in persons with DAT^54^ - Does not include the consequences of agitation on other residents and/or caregivers^54^ - Rates the duration and intensity of all 7 items from 0-3^54^ |

†**NR = Not Reported**

**Appendix 8: The Search Carried Out in MEDLINE from inception to August 14, 2021**

| 1 | Valid*.kf,tw. | 696503 |
| --- | --- | --- |
|  |  |  |
| 2 | screen*.kf,tw. | 718121 |
|  |  |  |
| 3 | tool*.kf,tw. | 685254 |
|  |  |  |
| 4 | Questionnaire*.kf,tw. | 487480 |
|  |  |  |
| 5 | Diagnostic Accuracy*.kf,tw. | 41859 |
|  |  |  |
| 6 | Psychometric*.kf,tw. | 45580 |
|  |  |  |
| 7 | survey*.kf,tw. | 619103 |
|  |  |  |
| 8 | test*.kf,tw. | 3113502 |
|  |  |  |
| 9 | Instrument*.kf,tw. | 279381 |
|  |  |  |
| 10 | inventory*.kf,tw. | 76607 |
|  |  |  |
| 11 | sensitivity.kf,tw. | 770383 |
|  |  |  |
| 12 | Specificity*.kf,tw. | 448710 |
|  |  |  |
| 13 | neuropsychological tests*.kf,tw. | 8388 |
|  |  |  |
| 14 | exp screening/ | 124176 |
|  |  |  |
| 15 | exp Mass Screening/ | 124176 |
|  |  |  |
| 16 | exp ROC Curve/ | 54651 |
|  |  |  |
| 17 | exp sensitivity/ and specificity/ | 340764 |
|  |  |  |
| 18 | exp questionnaires/ | 989566 |
|  |  |  |
| 19 | exp psychometrics/ | 72785 |
|  |  |  |
| 20 | exp neuropsychological test/ | 172975 |
|  |  |  |
| 21 | exp clinical decision making/ | 7305 |
|  |  |  |
| 22 | 1 or 2 or 3 or 4 or 5 or 6 or 7 or 8 or 9 or 10 or 11 or 12 or 13 or 14 or 15 or 16 or 17 or 18 or 19 or 20 or 21 | 6621116 |
|  |  |  |
| 23 | exp aggression/ | 37091 |
|  |  |  |
| 24 | exp Psychomotor agitation/ | 6256 |
|  |  |  |
| 25 | aggress*.kf,tw. | 200363 |
|  |  |  |
| 26 | Agitat*.kf,tw. | 19304 |
|  |  |  |
| 27 | 23 or 24 or 25 or 26 | 236930 |
|  |  |  |
| 28 | exp lewy body/ | 1751 |
|  |  |  |
| 29 | exp Dementia/ or exp Frontotemporal Dementia/ or exp Dementia, Vascular/ or exp Dementia, Multi-Infarct/ | 159429 |
|  |  |  |
| 30 | exp Alzheimer's Disease/ | 90213 |
|  |  |  |
| 31 | exp Parkinson's Disease/ | 63875 |
|  |  |  |
| 32 | (parkinson*s disease adj10 dementia).kf,tw. | 3938 |
|  |  |  |
| 33 | Alzheimer*.kf,tw. | 141169 |
|  |  |  |
| 34 | Lewy Bod*.kf,tw. | 8993 |
|  |  |  |
| 35 | neurocognitive*.kf,tw. | 19383 |
|  |  |  |
| 36 | frontotemporal*.kf,tw. | 10787 |
|  |  |  |
| 37 | dementia*.kf,tw. | 106626 |
|  |  |  |
| 38 | 28 or 29 or 30 or 31 or 32 or 33 or 34 or 35 or 36 or 37 | 321278 |
|  |  |  |
| 39 | 22 and 27 and 38 | 2576 |
|  |  |  |
| 40 | exp Human immunodeficiency virus/ | 96843 |
|  |  |  |
| 41 | (HIV or Human immunodeficiency virus).kf,tw. | 323206 |
|  |  |  |
| 42 | 40 or 41 | 330563 |
|  |  |  |
| 43 | 39 not 42 | 2560 |
|  |  |  |
| 44 | exp child/ | 1863949 |
|  |  |  |
| 45 | 43 not 44 | 2506 |
|  |  |  |
| 46 | exp animal/ | 22778245 |
|  |  |  |
| 47 | exp human/ | 18132015 |
|  |  |  |
| 48 | 46 not 47 | 4646230 |
|  |  |  |
| 49 | exp letter/ | 1053930 |
|  |  |  |
| 50 | exp editorial/ | 510697 |
|  |  |  |
| 51 | 49 or 50 | 1564452 |
|  |  |  |
| 52 | 45 not 48 | 2461 |
|  |  |  |
| 53 | 52 not 51 | 2441 |

**Appendix 9: Additional Diagnostic Accuracy Measures of Agitation and/or Aggression Diagnostic Tools used within the Dementia Population among Included Studies that compared tools to a reference standard**

| Author | Year | ROC of agitation tool | ROC of aggression tool | Combined ROC of agitation and aggression tool | PPV of agitation tool | PPV of aggression tool | NPV of agitation tool | NPV of aggression tool |
| --- | --- | --- | --- | --- | --- | --- | --- | --- |
| Ismail et al. | 2013 | E-BEHAVE-AD: 0.822 |  | E-BEHAVE-AD: 0.822 | NR | NR | NR | NR |
|  |  | NBRS: 0.900 |  | NBRS: 0.900 |  |  |  |  |
|  |  | NPI: 0.839 |  | NPI: 0.839 |  |  |  |  |
| Adama et al. | 2013 | N/A | 0.96 | 0.96 | N/A | 96% | N/A | 86% |
| Vilalta-Franch et al. | 1999 | NR |  | NR | NR |  | NR |  |
| Rosen et al. | 1999 | PAS: 0.85  NBRS: 0.77 |  | PAS: 0.85  NBRS: 0.77 | NR | NR | NR | NR |
| Sano et al. | 2022 | NR |  | NR | 32.3% |  | 94.7% |  |

†**NR= not reported; ROC= Receiver Operating Characteristic Curve; PPV= positive predictive value; NPV = negative predictive value**

**Appendix 10: PRISMA 2020 Checklist^58^**

| **Section and Topic** | **Item #** | **Checklist item** | **Reported on Page:** |
| --- | --- | --- | --- |
| **TITLE** | | |  |
| Title | 1 | Identify the report as a systematic review. | 1 |
| **ABSTRACT** | | |  |
| Abstract | 2 | See the PRISMA 2020 for Abstracts checklist. | 3 |
| **INTRODUCTION** | | |  |
| Rationale | 3 | Describe the rationale for the review in the context of existing knowledge. | 4 |
| Objectives | 4 | Provide an explicit statement of the objective(s) or question(s) the review addresses. | 5 |
| **METHODS** | | |  |
| Eligibility criteria | 5 | Specify the inclusion and exclusion criteria for the review and how studies were grouped for the syntheses. | 6/Figure 1 |
| Information sources | 6 | Specify all databases, registers, websites, organisations, reference lists and other sources searched or consulted to identify studies. Specify the date when each source was last searched or consulted. | 6/ Appendix 2 |
| Search strategy | 7 | Present the full search strategies for all databases, registers and websites, including any filters and limits used. | Appendix 1 |
| Selection process | 8 | Specify the methods used to decide whether a study met the inclusion criteria of the review, including how many reviewers screened each record and each report retrieved, whether they worked independently, and if applicable, details of automation tools used in the process. | 7 |
| Data collection process | 9 | Specify the methods used to collect data from reports, including how many reviewers collected data from each report, whether they worked independently, any processes for obtaining or confirming data from study investigators, and if applicable, details of automation tools used in the process. | 7-8 |
| Data items | 10a | List and define all outcomes for which data were sought. Specify whether all results that were compatible with each outcome domain in each study were sought (e.g. for all measures, time points, analyses), and if not, the methods used to decide which results to collect. | 7-8 |
|  | 10b | List and define all other variables for which data were sought (e.g. participant and intervention characteristics, funding sources). Describe any assumptions made about any missing or unclear information. | 5-8 |
| Study risk of bias assessment | 11 | Specify the methods used to assess risk of bias in the included studies, including details of the tool(s) used, how many reviewers assessed each study and whether they worked independently, and if applicable, details of automation tools used in the process. | 8 |
| Effect measures | 12 | Specify for each outcome the effect measure(s) (e.g. risk ratio, mean difference) used in the synthesis or presentation of results. | NA |
| Synthesis methods | 13a | Describe the processes used to decide which studies were eligible for each synthesis (e.g. tabulating the study intervention characteristics and comparing against the planned groups for each synthesis (item #5)). | 5-8 |
|  | 13b | Describe any methods required to prepare the data for presentation or synthesis, such as handling of missing summary statistics, or data conversions. | 8 |
|  | 13c | Describe any methods used to tabulate or visually display results of individual studies and syntheses. | 8 |
|  | 13d | Describe any methods used to synthesize results and provide a rationale for the choice(s). If meta-analysis was performed, describe the model(s), method(s) to identify the presence and extent of statistical heterogeneity, and software package(s) used. | 8 |
|  | 13e | Describe any methods used to explore possible causes of heterogeneity among study results (e.g. subgroup analysis, meta-regression). | NA |
|  | 13f | Describe any sensitivity analyses conducted to assess robustness of the synthesized results. | NA |
| Reporting bias assessment | 14 | Describe any methods used to assess risk of bias due to missing results in a synthesis (arising from reporting biases). | 8 |
| Certainty assessment | 15 | Describe any methods used to assess certainty (or confidence) in the body of evidence for an outcome. | 7-8 |
